# Two novel disease-causing mutations in the *CLRN1* gene in patients with Usher syndrome type 3

**Published:** 2012-12-29

**Authors:** Gema García-García, María J. Aparisi, Regina Rodrigo, María D. Sequedo, Carmen Espinós, Jordi Rosell, José L. Olea, M. Paz Mendívil, María A Ramos-Arroyo, Carmen Ayuso, Teresa Jaijo, Elena Aller, José M. Millán

**Affiliations:** 1 Grupo de Investigación en Enfermedades Neurosensoriales. Instituto de Investigación Sanitaria IIS - La Fe. Valencia, Spain; 2 Centro de Investigación Biomédica en Red de Enfermedades Raras (CIBERER), Valencia, Spain; 3 Servicio de Genética. Hospital Universitario Son Espases. Palma de Mallorca, Spain; 4 Servicio de Oftalmología. Hospital Universitario Son Espases. Palma de Mallorca, Spain; 5 Servicio de Oftalmología, Hospital de Basurto, Bilbao, Spain; 6 Servicio de Genética. Hospital Virgen del Camino, Pamplona, Spain; 7 Servicio de Genética, Instituto de Investigacion Sanitaria-Fundacion Jimenez Diaz (IIS-FJD). Madrid, Spain; 8 Unidad de Genética. Hospital Universitario La Fe. Valencia, Spain

## Abstract

**Purpose:**

To identify the genetic defect in Spanish families with Usher syndrome (USH) and probable involvement of the *CLRN1* gene.

**Methods:**

DNA samples of the affected members of our cohort of USH families were tested using an USH genotyping array, and/or genotyped with polymorphic markers specific for the USH3A locus. Based on these previous analyses and clinical findings, *CLRN1* was directly sequenced in 17 patients susceptible to carrying mutations in this gene.

**Results:**

Microarray analysis revealed the previously reported mutation p.Y63X in two unrelated patients, one of them homozygous for the mutation. After *CLRN1* sequencing, we found two novel mutations, p.R207X and p.I168N. Both novel mutations segregated with the phenotype.

**Conclusions:**

To date, 18 mutations in *CLRN1* have been reported. In this work, we report two novel mutations and a third one previously identified in the Spanish USH sample. The prevalence of *CLRN1* among our patients with USH is low.

## Introduction

Usher syndrome (USH) is an autosomal recessive disease characterized by the association of hearing loss and visual impairment due to retinitis pigmentosa (RP), with or without vestibular dysfunction. USH is the most frequent cause of concurrent deafness and blindness of genetic origin, and general prevalence ranges from 3.3 to 6.4 per 100,000 live births. However, recent studies indicate that the prevalence might be as high as 1 per 6,000 [1,2].

USH is clinically and genetically heterogeneous. Three clinical forms have been distinguished: USH1, USH2, and USH3. Nine genes have been identified responsible so far [3,4]. Five causative genes have been reported for USH1: *MYO7A*, *USH1C*, *CDH23*, *PCDH15*, and *USH1G*. Three genes have been described for USH2 (*USH2A*, *GPR98*, and *DFNB31*), and one gene has been described for USH3: *CLRN1.* There is growing evidence suggesting that at least eight of these proteins (all except clarin 1) form a network, which is critical for developing and maintaining the sensorineural cells in the inner ear and the retina [5-7].

The *CLRN1* gene is complex. At least 11 splice variants have been reported [8]. The main splice variant is composed of three exons that code for a 232 amino acid protein, clarin 1. Clarin 1 is a four-transmembrane protein expressed in the hair cells of the organ of Corti and in the neural retina [9,10].

The clarin 1 protein is thought to be expressed in mouse cochlea transiently from embryonic day 18 (E18) to postnatal day 6 (P6) in basal parts of the hair cells, whereas in apical parts (stereocilia) clarin 1 expression is lost already at P1. In the adult mouse retina, clarin 1 localizes to inner segments, connecting cilia and ribbon synapses. The function of clarin 1 remains unknown; however, the spatiotemporal expression pattern of clarin 1 in hair cells implicates protein involvement in synaptic maturation [11,12]. Structural and sequence homology with the synaptic protein stargazin suggest a role for clarin 1 in the plasma membranes surrounding the ribbon synapses of the inner ear and retina transport [9].

USH3 is characterized by progressive hearing loss, retinitis pigmentosa, and variable vestibular dysfunction; the progressiveness of hearing loss is a distinctive feature of USH1 and USH2 [13]. Although the *CLRN11* gene was initially described as responsible for USH3 cases, recent studies have demonstrated that mutations in *CLRN1* are also seen in Usher clinical forms similar to USH1 and USH2 or even isolated RP [14-16].

Usher syndrome type 3 is rare except among Finns and Ashkenazi Jews. In fact, most studies show that USH3 represents less than 5% in the majority of populations. In Finland, at least 40% of patients with Usher syndrome display type 3 whereas USH1 accounts for 34% of cases, and USH2 represents only 12% [17]. Furthermore, most patients carry one of the two named “Finnish mutations.” The Finnish founder mutation, Finmajor, is the nonsense mutation c.528T>G at codon 176 (p.Y176X), and is responsible for 94% of the patients with USH3 studied [18]. The Finminor mutation is c.359T>A (p.M120K), which is responsible for virtually the rest of the *CLRN1* alleles.

Among Ashkenazi Jews, the single mutation c.144T>C (p.N48K) is responsible for approximately 40% of USH cases, which means virtually 100% of the USH3 cases in the Ashkenazi population [19]. Haplotype analysis suggests a founder effect and an ancestral origin for these three mutations.

**Figure 1:**
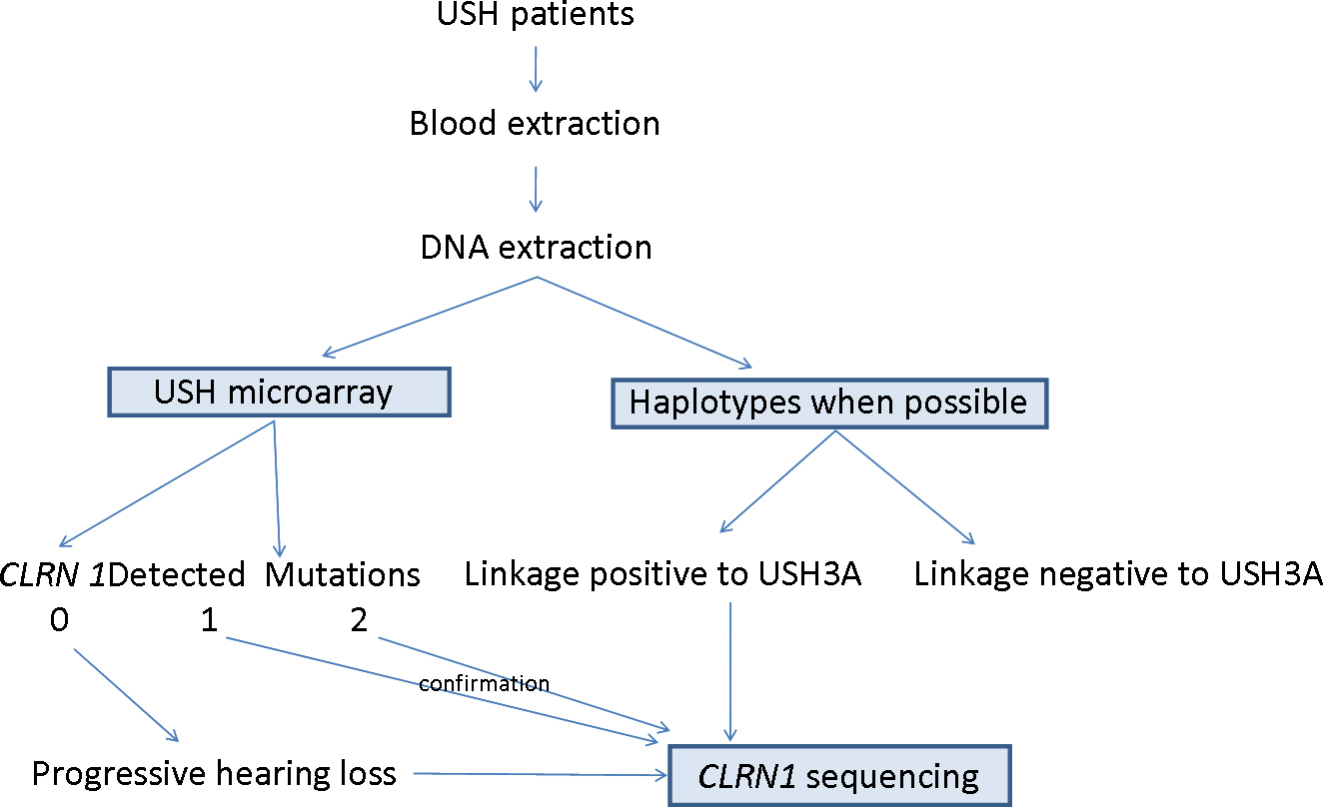
Algorithm for the study of USH3 in our series. When the family has more than one affected member or there is consanguinity, we haplotype the family to discard or not discard linkage to *CLRN1*. In parallel, we send DNA for USH microarray genotyping. If haplotypes are compatible with USH3A linkage or the USH microarray detects only one mutation, we perform direct sequencing of *CLRN1*. All the mutations detected with the USH microarray were confirmed with direct sequencing.

In this study, we sequenced the coding region of *CLRN1* in 17 unrelated patients with USH according to our algorithm for USH3 diagnosis (Figure 1). We found the mutation previously described p.Y63X [9] and two novel pathogenic mutations, p.R207X and p.I168N.

## Methods

### Subjects

Spanish subjects with Usher syndrome were mainly recruited from the Federación de Asociaciones de Afectados de Retinosis Pigmentaria del Estado Español (FARPE) and the Ophthalmology and ENT Services of several Spanish hospitals as part of a large study of the genetics of Usher syndrome in Spain. Informed consent approved by the Ethics Committee of the Hospital La Fe was obtained for all patients, and the study followed the tenets of the Declaration of Helsinki.

Subjects were classified as having USH based on their clinical history and underwent mutation screening of *CLRN1* as part of the algorithm for the molecular diagnosis of USH performed in our laboratory, which includes previous analysis with the Asper Biotech Usher genotyping microarray and linkage analysis and haplotype compatibility when possible. DNA from 50 individuals without a family history of hearing impairment or visual alterations were screened as healthy controls to evaluate the frequency of the mutations found in the patient sample.

### Clinical evaluation

Auditory function was assessed with otoscopy, standard pure tone audiometry (250–800 Hz), and tympanometry. In many cases, the localization of hearing loss in the cochlea was confirmed with otoacoustic emissions and auditory brainstem responses. Ophthalmological evaluations comprised funduscopy, a acuity test, a visual field test, and electroretinography. Vestibular evaluation was performed with a bithermal-caloric test, a rotatory test, computerized dynamic posturography, and a Romberg test.

### Microarray analysis

Genomic DNA was extractd from peripheral blood collected in EDTA tubes using an automated DNA extractor (MagNA Pure compact Instrument, Roche Applied Science, GmbH). Five micrograms of DNA were sent to Asper Biotech (Tartu, Estonia) for analysis with the USH microarray. This microarray detects 612 mutations from the nine USH genes [20].

The results obtained from the microarray were confirmed with direct sequencing. All exons where a mutation was identified were amplified with PCR. Amplicons were directly sequenced with dye termination chemistry (Prism Big Dye Terminator v1.1, Applied Biosystems, Inc. [ABI] Foster City, CA), and purified sequencing reactions were resolved in a sequencer (Prism 3130×l; ABI). To ascertain parental origin, segregation analysis was performed in cases in which at least two pathologic variants were identified.

### Construction of haplotypes

Haplotypes were constructed using the intragenic single nucleotide polymorphism (SNP) c.57A>T rs3796242 and two flanking extragenic markers D3S3661 and D3S1279 located upstream and downstream of *CLRN1*, respectively.

### Mutation screening

Exons 0, 2, and 3 (corresponding to the main isoform; NM_174878.2) and exon 1 (expressed in isoform NM_052995), including intron-exon boundaries of the *CLRN1* gene, were amplified using primers previously described by Adato et al. [9] with modifications and standard PCR conditions. Primers used for amplification of the four *CLRN1* exons are shown in Table 1.

**Table 1:** Primers used for amplification of the coding sequence of the CLRN1 gene.

Exon	Primers	Sequences (5′-3′)	Size (bp)	Primers described by
	0-D	TCCCATTGCTCACAAAGGTCTTGTTTTTG	380	Present study
0	0-R	ATTCCTCGCAACACTGGGAA	Present study	
1	1-D	TCACTATCTGAAACTATCTTGTTGT	910	9
	1-R	AAGCCCCTGAACTTTATAGG	9	
2	2-D	TCAGAAGGATTTTAGTGATGTTTGA	358	9
	2-R	AGACGGTCTTTTTGACATATTGAAAAGCACA	Present study	
3	3AB-D	ATGTCAATGGGGATGATGGT	981	9
	3AB-D-N	TTTACACATTGACCCTCTTCC	946	Present study
	3AB-R	CAGGCTGTAACTCGAACTCC	Present study	
	3B-D	GTAGCTGCAGATCTAATGTAC	242	Present study
	3B-R	GTCAAGCAATTTCCCACCAG	Present study	
	3BC-D	AAGTATACTCTTAGGCCAGGC	944	Present study
	3BC-R	CCTTTGTGGCTAGACTGAATT	Present study	

PCR products were sequenced using the manufacturer’s recommendations (Applied Biosystems). The sequences obtained were compared with the consensus genomic sequence NG_009168.1 using the BLAST program.

In cases where mutations were detected, we performed segregation analysis. To construct family trees, we used Cyrillic version 2.02 software.

### Predictions of the pathogenic effect of missense variations

To predict whether a rare missense variant is deleterious, we used the combined results of three computer algorithms: Sort Intolerant From Tolerant (SIFT) uses sequence homology to predict whether a change is tolerated or deleterious. The polymorphism phenotyping program PolyPhen uses sequence conservation, structure, and SWISS-PROT annotation to characterize an amino acid substitution as probably damaging, possibly damaging, benign, or unknown. Pmut provides prediction by neural networks, which use internal databases, secondary structure prediction, and sequence conservation. This program provides a binary prediction of “neutral” or “pathologic.”

## Results

### Mutation analysis

Previous studies identified two *CLRN1* mutations responsible for USH in two Spanish families. The mutation c.189C>A that led to a substitution of a tyrosin by a stop codon resulting in a truncated protein (p.Y63X) was found by Adato et al. in 2002 [21] in homozygous state in a family diagnosed with USH1. The second mutation (p.C40G) was homozygously found in a possible consanguineous Spanish USH3 family by Aller et al. in 2004 [22]. Both mutations were incorporated in the Usher genotyping microchip from Asper Ophthamics (Tartu, Estonia).

Further microchip analyses identified the p.Y63X mutation in homozygous state in the patient of another consanguineous family (FRP-44) and in heterozygous state in a patient from a second family (FRP-529).

**Figure 2:**
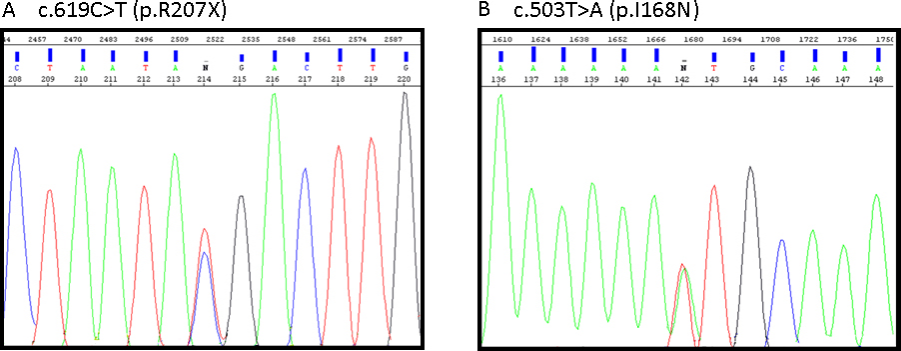
Electropherograms showing the novel *CLRN1* mutations identified in this study. **A**: c.619C>T mutation (p.R207X). **B**: c.503T>A mutation (p.I168N).

The screening of the *CLRN1* gene in the affected woman of FRP-529 revealed a novel nonsense mutation in exon 3, c.619C>T, that led to a truncated protein in codon 207, p.R207X. Figure 2A shows the electropherogram of the sequence showing the c.619C>T mutation. The segregation analysis revealed that the mother was the carrier of the novel mutation p.R207X whereas the father was the carrier of p.Y63X. The healthy sib was a carrier of p.Y63X.

Haplotype analysis in the family FRP-360 was compatible with disease association with the USH3A locus. After *CLRN1* mutation screening was conducted in this family, the c.619C>T (p.R207X) mutation was detected heterozygously in both affected men together with the missense mutation c.503T>A (p.I168N). Figure 2B shows the electropherogram corresponding to the c.503T>A mutation.

No other pathological mutations were found in *CLRN1* in any of the additional 15 USH samples. Table 2 shows the mutations in *CLRN1* found in the Spanish USH population detected in this and previous studies.

**Table 2:** Mutations identified in the CLRN1 gene in the Spanish USH population.

Nucleotide change	Exon	Predicted effect	No. of alleles	No. of families	References
c.118C>T	0	p.C40G	2	1	[22]
c.189C>A	0	p.Y63X	9	3	[9]
**c.503T>A**	**3**	**p.I168N**	1	1	Present Study
**c.619C>T**	**3**	**p.R207X**	2	2	Present Study

The novel mutations detected in this study are marked in bold. The reference sequence for nomenclature is NM_174878.2. Column 4 indicates the number of mutated alleles in the total Spanish USH series. Column 5 indicates the number of families in which the mutation was detected.

### In silico prediction

We performed computational analysis with the programs SIFT, PolyPhen, and Pmut to infer the pathologic effect of the variant p.I168N. These programs generated a pathogenic nature prediction for the change. The SIFT program predicted that the substitution of an isoleucine for an asparagine in codon 168 (p.I168N) of the protein would affect its function (score of 0.00; normalized probabilities less than 0.05 are predicted to be deleterious; those greater than or equal to 0.05 are predicted to be tolerated). The program Pmut predicted that change in p.I168N was pathologic with a reliability of 0.9485. Finally, the program PolyPhen predicted that the amino acid substitution probably damaged the protein.

### Clinical description

The patients included in this study displayed a wide range of ophthalmological and audiological symptoms even among patients from the same family. Patient RP-614 from family FRP-44 carried the p.Y63X homozygously. She was the daughter of a consanguineous marriage. Her hearing loss was prelingual and severe. The patient used audiphones until the age of 33 when she underwent a cochlear implantation. Hearing loss appeared to be progressive. The onset of the visual phenotype occurred when she was 14 years old but progressed rapidly to severe RP. No data about vestibular function were available.

**Figure 3:**
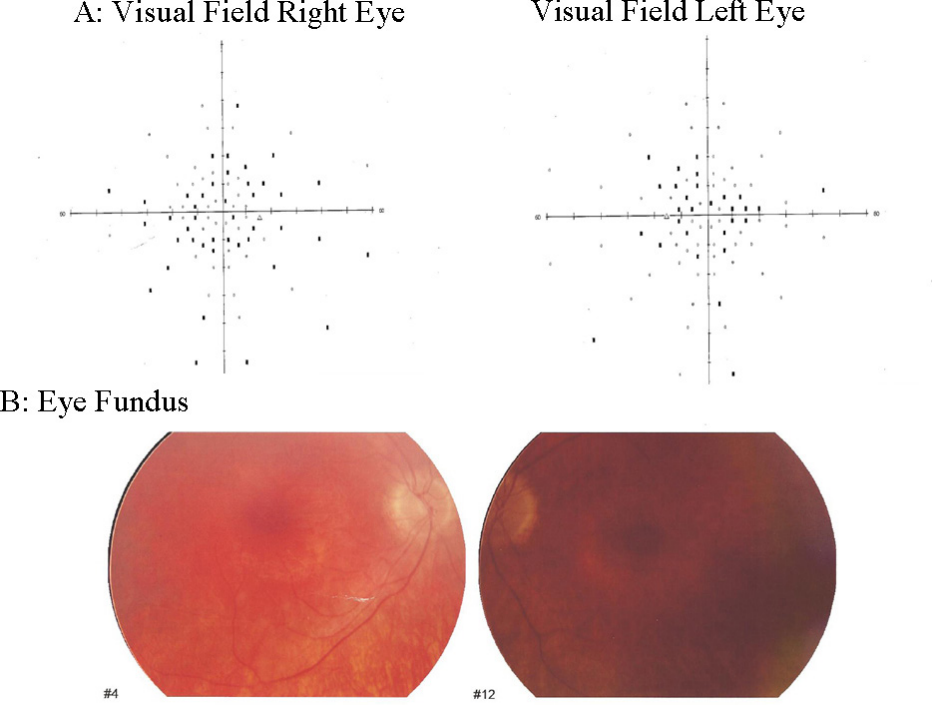
Ophthalmological features of the patient compound heterozygote for p.Y63X and p.R207X. **A**: Visual field, right eye and visual field, left eye. **B**: Eye fundus photography.

Patient RP-1850 from FRP-259 carried the p.Y63X mutation together with the novel p.R207X. She was a 19-year-old woman who presented with a bilateral severe progressive sensorineural hearing loss corrected with audiphones. Currently, she is waiting for cochlear implantation. She showed a delay in gait development and vestibular hyporeflexia. The onset of RP when she was 9 years old included night blindness and peripheral visual loss. Fundus ophthalmoscopy showed pigmentary anomalies typical of RP since she was young, attenuated arteriolar vessels and increased bright of the internal limitant membrane. The visual field showed an anular scotoma reduced by 15°. Her best-corrected visual acuity was 0.4 in both eyes, but she experienced a rapid progression of visual loss in the last year. The eye fundus and visual field of the patient are shown in Figure 3.

The patients from FRP-360 were compound heterozygotes for p.R207X and p.I168N and displayed discordant phenotypes. Patient RP-1520 is a 52-year-old man who started with night visual alterations at 6 years old, but was not diagnosed until he was 15. Visual acuity was 0.05 in both eyes, and he presented with pseudophakia. The eye fundus was typical of RP, and the visual field was reduced by 5° around the fixation point in both eyes. He had normal speech acquisition and normal motor milestones (he started gait at 9 months old). At 13 years old, he presented with a progressive hearing loss, without tinnitus or balance dysfunction. He needed audiphones at 42 years old, and currently he shows a moderate bilateral sensorineural hearing loss in the range of 79–80 dB and normal vestibular function.

Patient RP-1521 is a 49-year-old man who presented with severe hearing loss since he was born. He showed a delay in speech acquisition, starting to speak at 36 months and requiring deaf school education. He has had subjective symptoms of vestibular dysfunction since he was 10 years old. The first ophthalmological symptoms were detected at the age of 8 years old as night blindness and tunnel vision. The eye fundus was typical of RP with a reduction of 10° in OD and 20° in OI. The visual acuity was 0.2 (OD) and 0.3 (OI). A brief clinical description of the patients with mutations in *CLRN1* is shown in Appendix 1.

## Discussion

A total of 19 *CLRN1* mutations have been documented in patients with USH or RP around the world (Human Gene Mutation Database; Leiden Open Variation Database for Usher Syndrome). With the exception of the mutations p.Y176X and p.M120K found in the Finnish population [18] and p.N48K found in Ashkenazi Jews [9], the other mutations were mostly found in a single family.

In addition to these three common mutations, eight indels, one nonsense, and seven missense mutations have been reported to date: a deletion c.459_461delATT [18], a gross deletion of 23 bp (c.187_209del23bp) [9], a c.165delC deletion [10], a small indel mutation c.149_152delCAGGinsTGTCCAAT [10], a c.149_152delCAGG [10], a c.181delA [23], a c.301_305delGTCAT [24] and a c.502dupA insertion [25], a p.Y63X stop mutation [9], the c.161T>C (p.L54P) [26], the c.449T>C (p.L150P) [10], the c.118T>G (p.C40G) [22], the c.313T>C (p.S105P) [16] and the c.368C>A (p.A123D) [27], Finally, Khan et al. [28] found two homozygous missense mutations, c.92C>T (p.P31L) and c.461T>G (p.L154W), in two consanguineous Pakistani families with isolated RP and no audiological symptoms.

Two of the mutations found in this study are truncating: the p.Y63X mutation, found in two unrelated families who were carriers of Catalonian origin, and p.R207X, found in heterozygous state in two unrelated families who were carriers of Basque origin. The other mutation, p.I168N, is a missense and was found in a family together with the p.R207X mutation.

The physiologic consequences of the p.R207X and p.I168N mutations are unknown. Although *CLRN1* was identified in 2002, little is known about the protein, its function, or its domains. Clarin 1 is a membrane protein with four transmembrane domains that belongs to the family of vertebrate tetraspanins. The p.I168N mutation is located in the second extracellular loop. This mutation affects an isoleucine residue, hydrophobic and aliphatic substituted by the polar hydrophilic asparagine. To date, only two mutations have been described in the second extracellular loop, and they lead to truncated proteins. Thus, p.I168N could impair clarin 1 in some way, perhaps by altering the trafficking of the protein from the endoplasmic reticulum to the plasma membrane as has been observed for other missense mutations [28]. The mutation p.R207X leads to a clarin 1 that lacks the C-terminal domain. This C-terminal has a tobacco mosaic virus signature and could serve as a protein binding motif [9].

**Figure 4:**
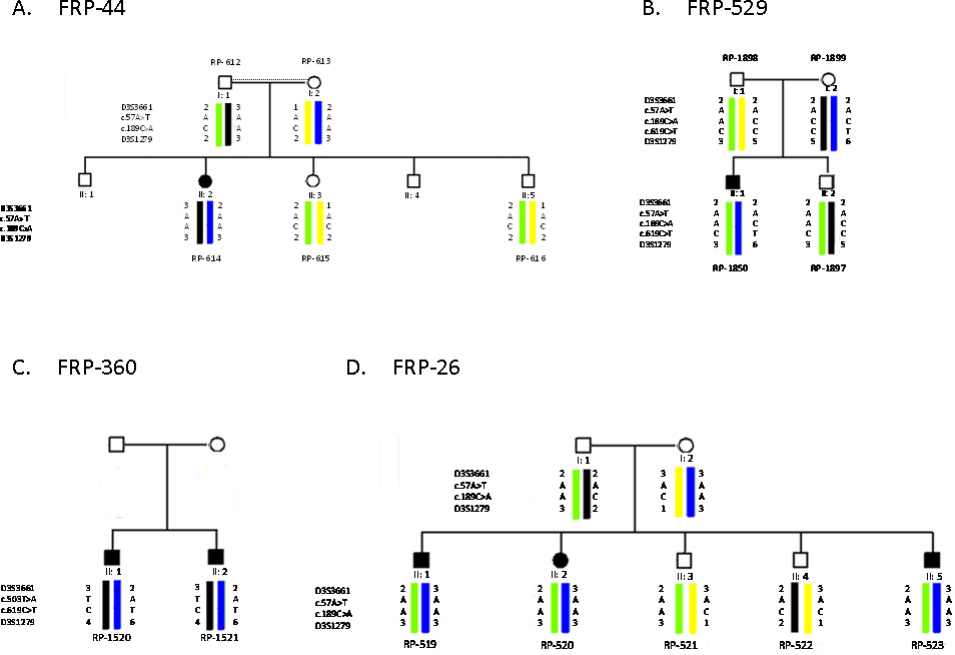
Pedigrees and haplotypes from four Spanish families with *CLRN1* mutations. **A**: Patient homozygote for c.189C>A (p.Y63X). **B**: Patient compound heterozygote for c.189C>A (p.Y63X) and c.619C>T (p.R207X). **C**: Patients compound heterozygotes for c.619C>T (p.Y63X) and c.503T>A (p.I168N). **D**: Patients homozygotes for c.169C>A (p.Y63X) initially described in [9].

In the total Spanish series, the mutation p.Y63X was observed in nine alleles of affected patients from three different families. The p.R207X mutation was detected in three alleles of affected patients from two families. A common haplotype was seen for p.R207X but not for p.Y63X (Figure 4).

The classical ophthalmological features of USH3 were defined by Joensuu [13] as night blindness, mid-peripheral visual field defects, and slowly progressive tunnel function vision at a mean age of 30 years. Fundus appearance was typical of RP with thin vessels and granular pigmentation in the midperiphery retina. The progression of RP resulted in a severe visual handicap at 20–30 years. The diagnosis of RP is usually made at the mean age of 17 years. Later, Plantiga et al. [29] found a visual acuity less than 0.05, severely impaired at mean age 37 years old, and tubular vision without peripheral islands at 30 years old. The visual deterioration rate was also similar to USH2A and USH1B, perhaps with more rapid progression in USH3A than USH2A, especially concerning visual field loss. The retinal degeneration pattern and structural changes in USH3A were similar to those in USH2A [30].

In the patients reported here, the mean age of diagnosis of RP was 11.5 years. Nevertheless, patient RP-1521 from family FRP-360 was diagnosed when he first noticed symptoms (8 years old), probably because his parents and the ophthalmologist were aware of the disease of his brother RP-1520. Visual acuity was poor except the younger patient, RP-1850, who is 19 years old, but she experienced a considerable loss of vision in the last year. All patients displayed night blindness and visual field loss as the first symptoms, and the eye fundus was typical of RP. Cataracts were present in two patients (RP-614 and RP-1520). RP-1850 has not developed cataracts at the age of 19 and neither has RP-1521 at the age of 50 years old while the elder brother has cataracts in both eyes.

Hearing loss was prelingual, severe, and progressive, and vestibular dysfunction was present except in patient RP-1520 who had postlingual moderate deafness with normal vestibular reflexes. These findings and especially the clinical features of the two affected sibs of FRP-360 illustrate the impressive wide spectrum of sensorineural hearing impairment in type and degree and the high degree of intersubject and intrafamiliar variability due to *CLRN1* mutations as previously reported [14].

The variable phenotype may cause USH3 to be underdiagnosed, and mutations in *CLRN1* might be more prevalent than previously thought. Nevertheless, the screening of *CLRN1* indicates that this gene is responsible for a low percentage of cases in our Spanish USH cohort.

## Appendix 1. Clinical description of patients in whom mutations in the CLRN1 gene were found.

To access the data, click or select the words “Appendix 1.” This will initiate the download of a compressed (pdf) archive that contains the file. Eye fundus: 1. Peripheral bone spicules, attenuated vessels, pale optic nerve · Visual field: normal: 0/ peripheral constriction or diffuse scotoma: 1/ anular scotoma or tubular field with islotes: 2/ Tubular central: 3/ islots or absolute scotoma: 4
